# Effect of EGFR amplification on the prognosis of EGFR-mutated advanced non–small-cell lung cancer patients: a prospective observational study

**DOI:** 10.1186/s12885-022-10390-0

**Published:** 2022-12-17

**Authors:** Duanyang Peng, Pingan Liang, Congying Zhong, Peng Xu, Yanqing He, Yuxi Luo, Xia Wang, Anwen Liu, Zhimin Zeng

**Affiliations:** 1 Department of Oncology, The Second Affiliated Hospital of Nanchang University, Nanchang University, Nanchang, Jiangxi Province PR China; 2 Jiangxi Key Laboratory of Clinical Translational Cancer Research, Nanchang, Jiangxi Province PR China; 3 Department of Nosocomial Infection Control, The Second Affiliated Hospital of Nanchang University, Nanchang, Jiangxi Province PR China; 4 Radiation Induced Heart Damage Institute of Nanchang University, Nanchang, Jiangxi Province PR China

**Keywords:** Non-small cell lung cancer, EGFR amplification, Tyrosine kinase inhibitors, Progression-free survival, EGFR mutation

## Abstract

**Background:**

Epidermal growth factor receptor (EGFR) amplification refers to the copy number increase of EGFR gene, and is often identified as a “bypass” way of Epidermal growth factor receptor Tyrosine kinase inhibitors (EGFR-TKI) resistance. We aimed to explore the effect of EGFR amplification on EGFR mutation treatment-naive advanced non-squamous non-small cell lung cancer (NSCLC) patients.

**Methods:**

We conducted a prospective observational study in single center, enrolling advanced non-squamous NSCLC patients receiving Tyrosine kinase inhibitors (TKIs) between March 3, 2019, and February 1, 2022. Next-generation sequencing (NGS) was used to detect genetic alterations in tumor tissue samples. Progression-free survival (PFS) curves were performed using the Kaplan-Meier method. Univariate and multivariate analyses were used to evaluate factors affecting the efficacy of TKIs.

**Results:**

A total of 117 treatment-naive advanced NSCLC patients were identified in this study. EGFR amplification was found in 22 of 117 (18.8%) patients with EGFR mutations. Of 22 patients with EGFR amplification, 10 patients harbored EGFR 19 del, 11 patients with 21-L858R. The median follow-up time was 22.47 months. The median PFS of the patients with or without EGFR amplification was 8.25 months and 10.67 months, respectively (log-rank test, *P* = 0.63). In multivariate analysis, EGFR amplification was not an independent prognosis factor for the patients receiving first-line TKIs [HR = 1.38, 95%CI (0.73–2.58), *P* = 0.321]. Subgroup analysis revealed that EGFR amplification is a risk factor for progression in the brain metastasis population. [HR = 2.28, 95%CI (1.01, 5.14), *P* = 0.047].

**Conclusion:**

EGFR amplification is not an independent prognosis factor for PFS in advanced non-squamous NSCLC patients receiving first-line TKIs. However, it is an independent risk factor for PFS in the brain metastasis population.

## Introduction

Epidermal growth factor receptor (EGFR) mutation is the most common gene alteration in NSCLC, which accounts for approximately 15% of the Caucasians and 50% of Eastern Asians [1]. EGFR-TKI is the standard first-line treatment for EGFR-mutated lung cancer patients [2–5]. At present, the third generation EFGR-TKIs, such as Osimertinib or Aumolertinib, significantly improve response rates, progression-free survival (PFS), and overall survival (OS) in lung cancer patients with EGFR mutations [6, 7], but the outcome of partial patients remains extremely poor.

Both patient-related and tumor-related factors could affect the efficacy and survival of EGFR-TKI treatment in lung cancer patients. Different types of EGFR mutations may have different responses to the EGFR-TKIs, such as patients with EGFR exon 19 deletion (19del) having a longer PFS compared to patients with EGFR exon 21 Leu858Arg (21-L858R) mutation [8–12]. Furthermore, other studies have found that patients with different Eastern Cooperative Oncology Group (ECOG) scores, gender, and smoking status show different responses to TKIs [8, 13, 14]. In addition, co-mutations of EGFR mutation lung adenocarcinoma patients may also influence the response to TKI treatment. It has been reported that co-occurring abnormalities including TP53, RB1, PTEN, ERBB2, and MET are unfavorable clinical prognosis factors for lung adenocarcinoma patients receiving first-line EGFR-TKIs [15–17].

EGFR amplification means an increase in the copy number of the EGFR gene, which is generally recognized as a “bypass” way of EGFR-TKI resistance [18, 19]. Previous studies reported that EGFR amplification occurred in about 9–64% NSCLC patients [20–23], and EGFR amplification also occurs in treatment-naive EGFR-mutated patients [15, 24–26]. A post hoc analysis of the Iressa Pan-Asia Study (IPASS) reported that EGFR amplification alone is not a predictive factor for patients receiving first-line EGFR-TKIs treatment [27]. A retrospective study suggested that EGFR amplification was a predictive factor for a better survival benefit from first-line TKIs treatment [26]. Ruiz-Patino A et al. discovered a significant difference in PFS and OS between EGFR amplification patients with 19del and 21-L858R [25]. However, Gao X et al. found that EGFR amplification is an independent poor factor in NSCLC patients with EGFR exon 20 insert receiving TKIs [24]. Meanwhile, a retrospective study found that EGFR amplification is an unfavorable factor when patients are treated with third-generation TKIs [15]. Therefore, it is not clear whether EGFR amplification is an independent prognosis factor in EGFR mutation patients receiving TKIs.

In this study, we conducted a prospective observational study to evaluate the relationship between EGFR amplification and PFS in EGFR-mutated NSCLC patients.

## Methods

### Study design

Advanced non-squamous NSCLC patients with EGFR mutations were consecutively collected between March 3, 2019 and February 1, 2022 at the Second Affiliated Hospital of Nanchang University. Inclusion criteria are as follows: (1) older than 18 years; (2) TNM IV stage; (3) patients with pathologically confirmed lung adenocarcinoma, adenosquamous carcinoma, or other non-specific pathologic types; and (4) EGFR mutation confirmed by NGS of tissue specimens. Those who had a history of additional malignancies, previous treatment, or had incomplete clinical data were excluded. Patients’ characteristics of demographic, genomic, and clinical, including age, sex, smoking, ECOG score, type of pathology, TNM stage, distant metastases status, EGFR mutation, T790M mutation, TP53 mutation, radiotherapy, and type of EGFR-TKIs, were collected from the hospital information system. This study conformed to the Declaration of Helsinki and was approved by the Institutional Ethics Committee of the Second Affiliated Hospital of Nanchang University.

### Next-generation sequencing (NGS) detection

Lung tissues were obtained from percutaneous lung biopsies or bronchoscopy, and fixed in the Department of Pathology using paraffin wax. Formalin-fixed paraffin-embedded (FFPE) tumor tissues were collected for genetic variation detection. Gene mutation analysis of FFPE tumor tissue was determined by capture single molecule amplification and resequencing technology(capSMART) for 31or 457 cancer-related genes (Berry Oncology Beijing, China). In a nutshell, genomic DNA from tumor tissue samples was extracted using the DNeasy Tissue kit (Qiagen). The concentration of the purified DNA was determined by the Qubit^R^ dsDNA HS Assay Kit (Life Technologies, Grand Island, NY, United States). DNA libraries were constructed as previously described and the target-enriched library was then paired end (PE) sequenced (2 × 150 bp) on the NovaSeq platform (Illumina) according to the manufacturer’s instructions with high, uniform median coverage (> 1000×) and assessed for base substitutions, short insertions and deletions, copy number alterations, and gene fusions/rearrangements [28]. We determined the log ratio of DoC for each target (tumor versus control) and then used the circular binary segmentation (CBS) algorithm to segment the log ratio profile into segments of equal copy number for copy number analysis of the normalized collection of somatic variations [29]. We extracted CNV genes from the CBS segments. At first, genes with less than five target (target number ≤ 4) were filtered out. Then, for each gene target, we calculated the segment value presenting the mean log ratio of all target of this segment. When the segment value was ≥ 0.35, we consider this target as a gain target gain. If the number of gain targets / all targets for this gene was ≥ 0.7, and then this gene was considered as a gene amplification. Finally, we used the all of the targets of this gene to calculate the average log ratio, and calculated the average copy number. We used the formula to calculate the mutation of copy number n_mut_ =VAF1ppCNt + CNn1-p [30]. While CNt means the tumor locus specific copy number, CNn represents the normal locus specific copy number(assumed to be 2), p is the tumor purity calculated by Facet [31] and VAF represents the variant allele frequency. EGFR amplification was defined as the copy number over 4.

### Treatment and follow-up procedures

All the patients were treated with TKIs-based regimens, including TKIs alone or TKIs combined Anlotinib. PFS was calculated from the date of the TKI treatment to disease progression or death due to any cause. Evaluation of treatment effects was conducted according to the Response Evaluation Criteria in Solid Tumors (RECIST) version 1.1. The last follow-up time was June 22, 2022. The median follow-up time was 22.47 months.

### Statistical analysis

All the continuous variables were expressed as mean ± standard deviation, and categorical variables were expressed as percentages. Independent sample t-tests and Fisher’s exact test were used for comparisons between the groups. Survive curves were drawn using the Kaplan-Meier method and compared by log-rank tests. Univariate and multivariate Cox proportional hazards models were used to evaluate independent predictive factors of each demographic, genomic, and clinical character associated with survival. We took relevant factors including age, ECOG score, TNM stage, bone metastasis, liver metastasis, lung metastases, pleural metastasis, EGFR mutation, T790M, TP53 mutation, radiotherapy, and type of TKIs into multivariate analysis. A multivariable stratified analysis adjusted with age, was performed as the sensitivity analysis to assess the effect of EGFR amplification in the subgroup patients, expressed as a hazard ratio with a 95% CI. Multivariable HRs for progression based on EGFR amplification status stratified by gender, brain metastasis, EGFR mutation, TP53 mutation, and TKI type with adjusted age. P values less than 0.05 were recognized as statistically significant. The statistical software packages R (version 3.6.3, http://www.R-project.org, The R Foundation) and Empower Stats (http://www.empowerstats.com, X&Y Solutions, Inc., Boston, MA) were used to analyze all data.

## Results

### Patient characteristics

**Fig. 1:**
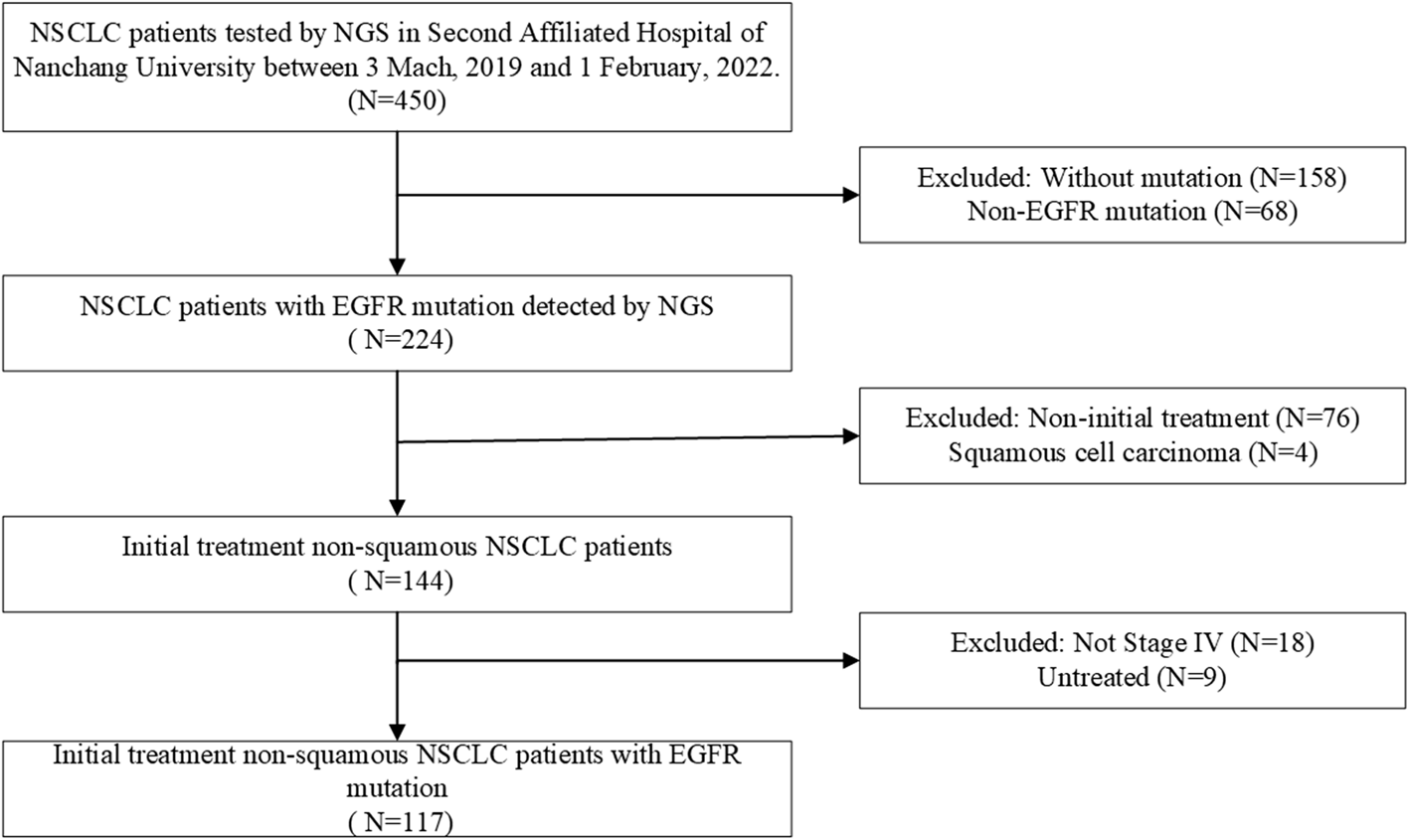
Flow chart of patient screening

A total of 117 non-squamous NSCLC patients with EGFR mutations were enrolled in our study, and eventually EGFR amplification was found in 22 of 117(18.8%) patients. The selection procedure was presented in Fig. 1. Table 1 showed that the enrolled patients included 70 females (59.83%), 88 patients (75.21%) aged under 70, and 16 patients (13.68%) with a smoking history. Seven patients (5.98%) received treatment combining first-generation EGFR-TKIs and Anlotinib, 65 patients (55.56%) were treated with the first-generation TKI, and 31patients (29.06%) were exposed to third-generation TKI.

**Table 1:** Characteristics of enrolled patients

Characteristic	EGFR-mutated	Without EGFR amplification	With EGFR amplification	*P* value
	N(%)	N(%)	N(%)	
Age				0.804
<70	88(74.21%)	71 (74.74%)	17 (77.27%)	
≥70	29(24.79%)	24 (25.26%)	5 (22.73%)	
Gender				0.064
Female	70(59.83%)	53 (55.79%)	17 (77.27%)	
Male	47(40.17%)	42 (44.21%)	5 (22.73%)	
ECOG score				0.944
0	22(18.81%)	18 (18.95%)	4 (18.18%)	
1	71(60.68%)	57 (60.00%)	14 (63.64%)	
2	24(20.51%)	20 (21.05%)	4 (18.18%)	
Smoking				0.167
No	101(86.32%)	80 (84.21%)	21 (95.45%)	
Yes	16(13.68%)	15 (15.79%)	1 (4.55%)	
Type of lung carcinoma				0.35
Adenocarcinoma	111(94.87%)	91 (95.79%)	20 (90.91%)	
Others (non-squamous NSCLC)	6(5.13%)	4 (4.21%)	2 (9.09%)	
TNM stage				0.604
IVA	27(23.08%)	21 (22.11%)	6 (27.27%)	
IVB	90(76.92%)	74 (77.89%)	16 (72.73%)	
Brain metastasis				0.755
No	62(52.99%)	51 (53.68%)	11 (50.00%)	
Yes	55(47.01%)	44 (46.32%)	11 (50.00%)	
Bone metastasis				0.865
No	46(39.32%)	37 (38.95%)	9 (40.91%)	
Yes	71(60.68%)	58 (61.05%)	13 (59.09%)	
Liver metastasis				0.277
No	104(88.89%)	83 (87.37%)	21 (95.45%)	
Yes	13(11.11%)	12 (12.63%)	1 (4.55%)	
Lung metastasis				0.778
No	66(56.41%)	53 (55.79%)	13 (59.09%)	
Yes	51(43.59%)	42 (44.21%)	9 (40.91%)	
Pleural metastasis				0.764
No	82(70.09%)	66 (69.47%)	16 (72.73%)	
Yes	35(19.91%)	29 (30.53%)	6 (27.27%)	
EGFR mutation				0.861
19del	49(41.88%)	39 (41.05%)	10 (45.45%)	
21-L858R	60(51.28%)	49 (51.58%)	11 (50.00%)	
other	8(6.84%)	7 (7.37%)	1 (4.55%)	
T790M (initial)				0.944
No	112(95.73%)	91 (95.79%)	21 (95.45%)	
Yes	5(4.27%)	4 (4.21%)	1 (4.55%)	
TP53 mutation				0.016
No	42(35.90%)	39 (41.05%)	3 (13.64%)	
Yes	75(64.10%)	56 (58.95%)	19 (86.36%)	
Radiotherapy				0.402
No	83(70.94%)	69 (72.63%)	14 (63.64%)	
Yes	34(29.06%)	26 (27.37%)	8 (36.36%)	
Initial treatment				0.548
1 generation TKI	65(55.56%)	55 (57.89%)	10 (45.45%)	
2 generation TKI	14(11.97%)	12 (12.63%)	2 (9.09%)	
3 generation TKI	31(26.49%)	23 (24.21%)	8 (36.36%)	
1 generation TKI + Anlotinib	7(5.98%)	5 (5.26%)	2 (9.09%)	

*Abbreviations*: *EGFR* epidermal growth receptor, *ECOG* Eastern Cooperative Oncology Group, *TNM* Tumor Node Metastasis, *19del* exon 19 deletion, *21-L858R* exon 21 Leu858Arg, *TKI* tyrosine kinase inhibitor

### Molecular characteristics

**Fig. 2:**
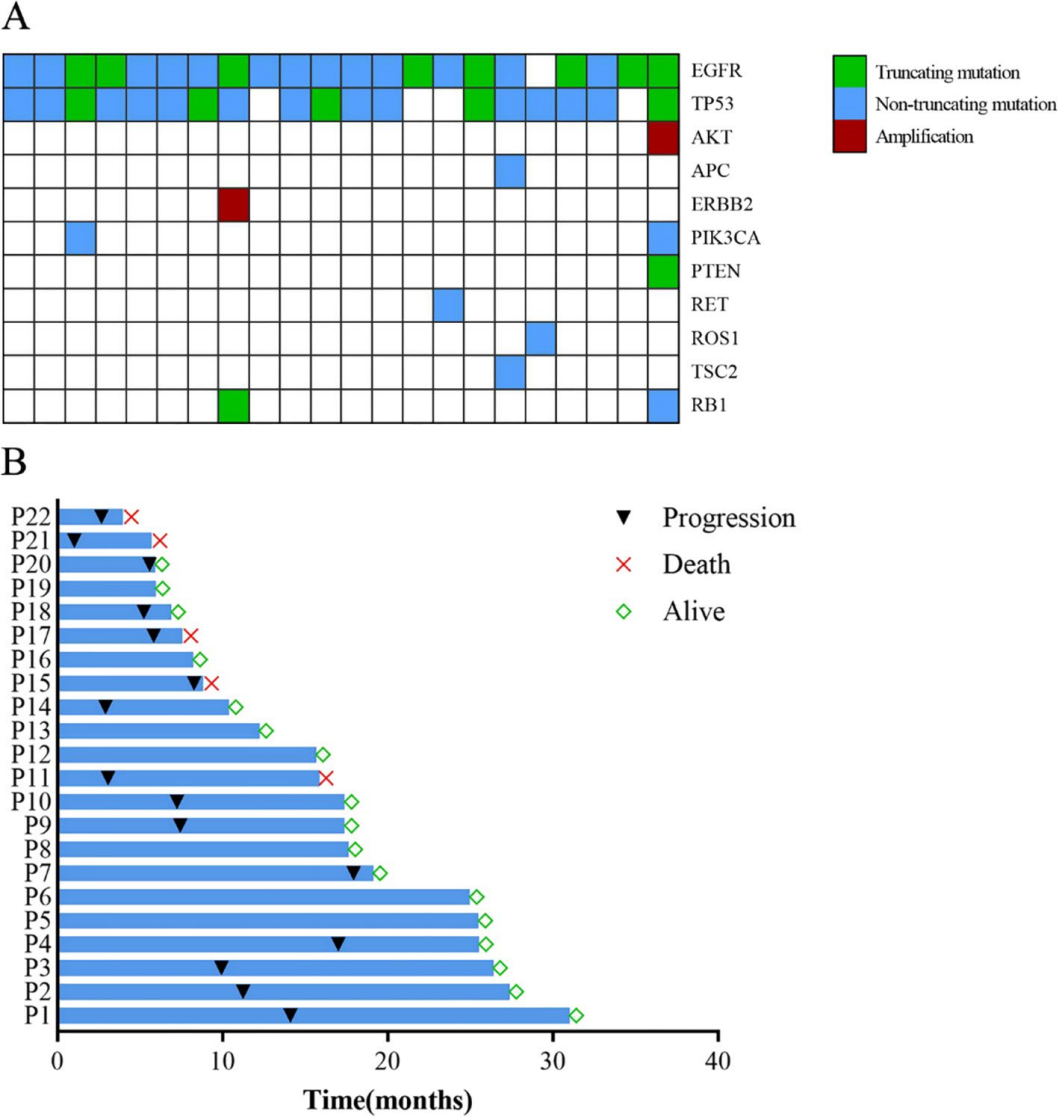
Gene distribution and the survival swimming plots of lung adenocarcinoma patients with EGFR mutation and EGFR amplification. **A **Gene distribution of lung adenocarcinoma patients with EGFR mutation and EGFR amplification; **B **survival swimming plots of lung adenocarcinoma patients with EGFR mutation and EGFR amplification

There were 49 (41.9%) patients harboring 19del, 60 (51.3%) with 21-L858R mutations, and 8 with uncommon EGFR mutations (2 patients with L861Q, 2 patients with I740_K745dupIPVAIK, 1 patient with 21p.L858_A859delinsRS, 1patent with G719X/V834L and 1 patient with G719X/A767V). Twenty-two EGFR amplification patients were detected with co-occurring alterations, including 10 (45.5%) EGFR19 del, 11(50%) 21-L858R. The detailed genomic alterations of patients with EGFR amplification were described in Fig. 2A. Table 1 showed that the proportion of females was higher in patients with EGFR amplification compared to those without (77.27% vs. 55.79%). Moreover, patients with EGFR amplification more often had co-mutation of TP53 compared with those without EGFR amplification (86.36% vs. 58.95%, *P* = 0.016). There were only 16 patients received NGS test again when the disease progresses. Of 16 patients, 1 patient carry MET amplification, 3 patients harboring T790M.

### Survival analysis

**Fig. 3:**
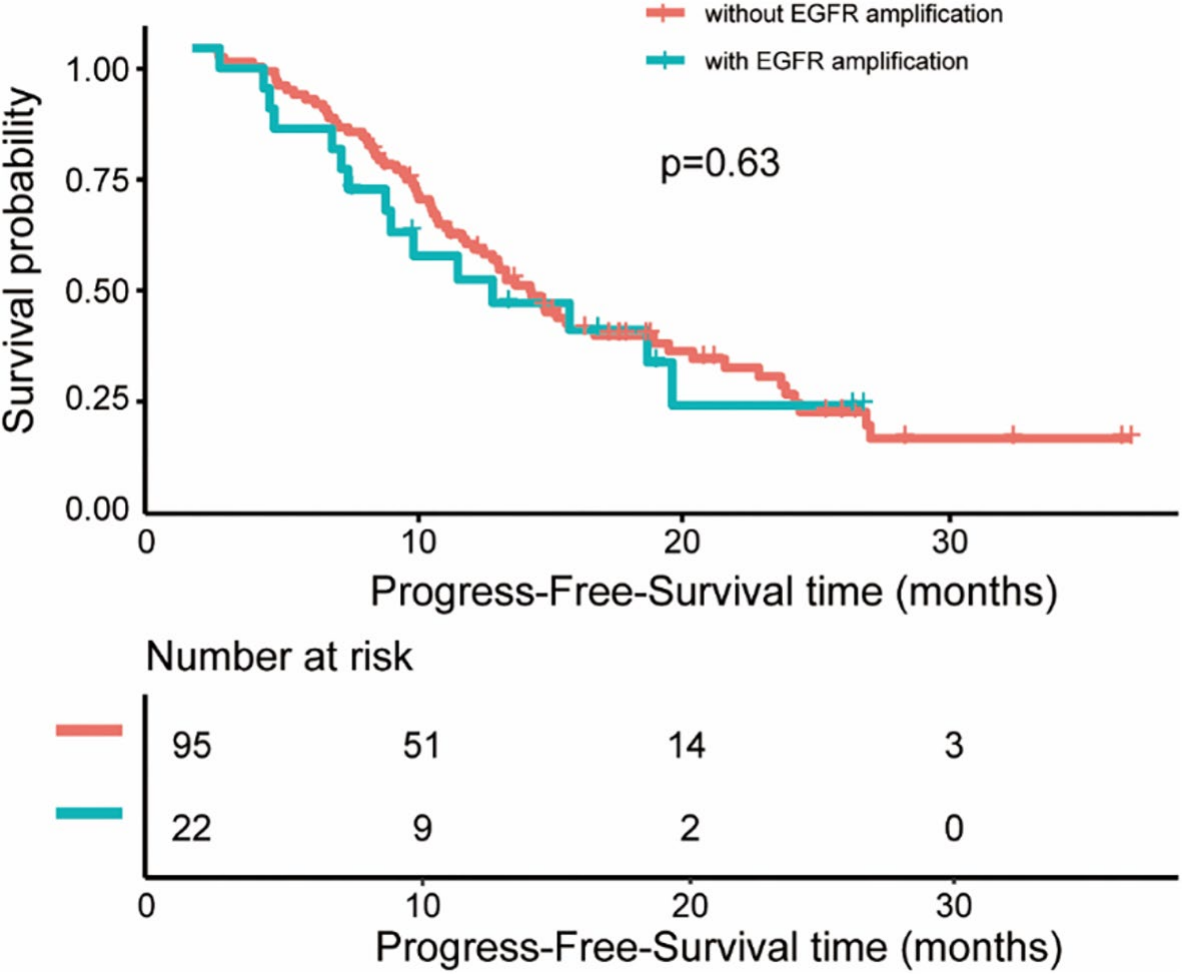
Progress-free survival curves of patients with EGFR amplification and without EGFR amplification

The median follow-up time was 22.47 months. The median PFS was 11.43 months, and 11.43 months for 19del, 11.23 months for 21-L858R, and 14.07 months for rare EGFR mutations in the entire group. Figure 2B displayed survival time of these 22 patients with EGFR amplification. The median PFS of patients with EGFR amplification and without EGFR amplification were 9.93months and 11.47 months respectively (*P* = 0.63, Fig. 3). Then, we conducted univariate analysis to evaluate factors affecting the efficacy of TKIs (Table 2). Univariate analysis showed that bone metastasis is associated with shorter PFS [HR = 1.58, 95%CI (1.01–2.50), *p* = 0.047]. In contrast, 3rd generation TKI is associated with favorable PFS [HR = 0.41, 95%CI (0.23, 0.73) *P* = 0,003]. However, EGFR amplification was not a favorable prognostic factor for PFS [HR = 1.15, 95%CI (0.66–2.01), *P* = 0.63] in univariate analysis. Multivariate analysis indicated that EGFR amplification was still not a favorable prognostic factor of PFS [HR = 1.38, 95% (0.73, 2.58), *P* = 0.321]. Stratified analysis was further conducted to assess the effect of EGFR amplification in the patient subgroups (Table 3), which revealed that EGFR amplification is a risk factor for PFS [HR = 2.28, 95%CI (1.01, 5.14), *P* = 0.469] in the brain metastasis population.

**Table 2:** Univariate and Multivariate Analyses progress-free survival prognosis of NSCLC EGFR-mutated patients

	Univariate analysis		Multivariate analysis	
	HR (95%CI)	*P* value	HR (95%CI)	*P* value
Age				
<70	1		1	
≥70	0.69(0.41,1.16)	0.1598	0.65 (0.34, 1.23)	0.1872
Gender				
female	1			
male	1.13(0.73, 1.75)	0.5869		
ECOG score				
0	1		1	
1	1.28 (0.73, 2.24)	0.3838	1.09 (0.59, 2.01)	0.7920
2	0.88 (0.42, 1.83)	0.3838	0.85 (0.38, 1.93)	0.6983
Smoking				
No	1			
Yes	0.85 (0.45, 1.61)	0.6175		
Type of pathological				
Adenocarcinoma	1			
Others	1.37 (0.50, 3.76)	0.5419		
TNM stage				
IVA	1		1	
IVB	1.62 (0.95, 2.77)	0.5419	1.29 (0.63, 2.65)	0.4792
Brain metastasis				
No	1			
Yes	1.15 (0.75, 1.77)	0.5156		
Bone metastasis				
No	1		1	
Yes	1.58 (1.01, 2.50)	0.0471	1.30 (0.71, 2.36)	0.3976
Liver metastasis				
No	1		1	
Yes	1.40 (0.74, 2.65)	0.2976	1.38 (0.65, 2.93)	0.3981
Lung metastasis				
No	1		1	
Yes	0.67 (0.43, 1.05)	0.2976	0.71 (0.43, 1.15)	0.1665
Pleural metastasis				
No	1		1	
Yes	1.29 (0.81, 2.05)	0.2897	1.34 (0.81, 2.21)	0.2551
EGFR mutation				
19del	1		1	
21-L858R	1.06 (0.68, 1.64)	0.8086	1.37 (0.82, 2.27)	0.2288
other	0.75 (0.27, 2.12)	0.5906	1.41 (0.40, 4.94)	0.2288
T790M (initial)				
No	1		1	
Yes	0.41 (0.13, 1.32)	0.1367	0.58 (0.16, 2.10)	0.4068
TP53 mutation				
No	1		1	
Yes	1.18 (0.75, 1.87)	0.4664	0.94 (0.56, 1.60)	0.8298
Radiotherapy				
No	1		1	
Yes	1.27 (0.80, 2.01)	0.4664	1.08 (0.62, 1.87)	0.7893
Initial treatment				
1 generation TKI	1		1	
2 generation TKI	0.93 (0.47, 1.83)	0.8289	0.64 (0.28, 1.46)	0.2927
3 generation TKI	0.41 (0.23, 0.73)	0.0026	0.39 (0.20, 0.77)	**0.0063**
1 generation TKI+ Anlotinib	0.64 (0.26, 1.61)	0.8289	0.54 (0.20, 1.44)	0.2183
EGFR amplification				
No	1		1	
Yes	1.15 (0.66, 2.01)	0.6300	1.38 (0.73, 2.58)	0.3210

*Abbreviations: CI* confidence interval, *HR* hazard ratio, *EGFR* epidermal growth receptor, *ECOG* Eastern Cooperative Oncology Group, *TNM* Tumor Node Metastasis, *19del* exon 19 deletion, *21-L858R* exon 21 Leu858Arg, *TKI* tyrosine kinase inhibitor, bold was considered significant

**Table 3:** Subgroup Analyses of progress-free survival prognosis of NSCLC EGFR-mutated patients

Factor	N	HR (95% CI)	*P* value
Gender			
Female	70	1.06 (0.54, 2.09)	0.8586
Male	47	2.17 (0.69, 6.85) 0.1862	0.1862
Brain metastasis			
No	62	0.82 (0.36, 1.86)	0.6417
Yes	55	2.28 (1.01, 5.14)	**0.0469**
EGFR mutation			
19del	49	1.28 (0.56, 2.97)	0.5588
21-L858R	60	0.92 (0.40, 2.11)	0.8510
Other	8	1.41 (0.08, 23.57)	0.8092
TP53 mutation			
Yes	42	2.90 (0.62, 13.66)	0.1777
No	75	1.00 (0.53, 1.89)	0.9985
Initial treatment			
1 generation TKI	65	1.07 (0.50, 2.28)	0.8580
2 generation TKI	14	1.89 (0.33, 10.74)	0.4732
3 generation TKI	31	2.07 (0.62, 6.89)	0.2335
1 generation TKI + Anlotinib	7	3.46 (0.22, 55.78)	0.3809

*Abbreviations: CI* confidence interval, *HR* hazard ratio, *EGFR* epidermal growth receptor, *19del* exon 19 deletion, *21-L858R* exon 21 Leu858Arg, *TKI* tyrosine kinase inhibitor. bold was considered significant

## Discussion

To the best of our knowledge, this is the first prospective observational cohort study evaluating the EGFR amplification prognostic value in EGFR-mutated NSCLC patients treated with first-line TKIs. We identified a total of 22 (18.8%) patients with EGFR amplification. Patients with EGFR amplification often have TP53 mutations. Our results revealed that concomitant concurrence of EGFR amplification is not associated with longer PFS in patients receiving first-line EGFR-TKIs. But subgroup analysis revealed that EGFR amplification is a risk factor for progression in patients with brain metastasis.

The proportion of EGFR amplification in our study was lower than previous studies, which reported EGFR amplification appeared in approximately 30-47% EGFR-mutated NSCLC patients [24–26]. The discrepancy might derive from sample size and methods of detecting EGFR amplification. In the study conducted by Ling Shana et al. and Ruiz-Patino A et al., they used the Dual-color Silver in situ Hybridization (DISH) or the fluorescence in situ hybridization (FISH) to detect EGFR amplification [25, 26]. Consistent with us, a study about EGFR 20 insert also used NGS to examine EGFR amplification, the definition of EGFR amplification was gene copy number > 2.75 in the 520 panel and > 2.25 in the other panels [24]. But in our study, EGFR amplification was defined as a copy number greater than 4 regardless of the small or large panels. Among the assay methods of NGS, there is not a consensus definition of EGFR amplification [32, 33]. Shan L et al. firstly demonstrated that EGFR amplification can influence treatment effects for NSCLC EGFR-mutated patients receiving TKIs [26]. The study among Hispanic patients also identified EGFR amplification as a prognosis factor for containing EGFR mutation patients treated with erlotinib, and further suggested that there is a significant difference between EGFR amplification patients with 19del and 21-L858R no matter in PFS or OS [25]. But our results suggest that co-occurring EGFR amplification is not a prognosis factor for patients treated with first-line TKIs, and we did not find any significant difference between patients with 19del and 21-L858R in PFS. Several reasons might account for the discrepancy. Firstly, both studies by Ling Shana and Ruiz-Patino A et al. were retrospective, while our study was a prospective observational study. In addition, these two did not include the other potentially coexisting factors, such as TP53 mutation and radiotherapy, which might influence the therapeutic efficacy of TKIs. Moreover, our study included different types of TKIs while enrolled patients were treated with 1st generation TKIs in their studies. Another study used a novel method to assess the heterogeneity of EGFR copy number gain [22]. Their results indicated that the EGFR copy number is significantly heterogeneous in different NSCLC patients. This discovery may help to explain the conflicting clinical data on EGFR amplification.

In addition to EGFR amplification, lots of studies have identified co-alterations including TP53 as a negative prognostic factor for EGFR-mutated NSCLC and a consistent predictor of poor survival outcome of EGFR-mutated patients receiving TKIs treatment [15–17, 34]. Paolo Bironzo et al. find that co-mutations including TP53 are not a predictive factor for patients treated with first-line TKIs [35]. However, we failed to identify TP53 as a prognosis factor. The main reasons might come from the size of the sample and heterogeneity of the included study population and usage of different EGFR-TKIs. Consistent with the study conducted by Bironzo P et al., the third-generation TKI was an independent predictor for patients with EGFR mutations [35]. Their study was also a single institution study based on real world data, including patients treated with first, second, and third generation TKIs. Both study of Bironzo P et al. and us are small sample studies, while our studies was prospective.

It has been established that EGFR copy gain is related to activating mutations only at the mutated oncogene locus but not in other oncogene loci [36, 37]. It is one of the typical examples that mutant allele specific imbalance caused by copy number gain or uniparental disomy. It frequently occurs in an important subset of cells containing mutant oncogenes due to complete loss of wild type allele [36, 38]. Besides, a growing body of research indicated that EGFR mutations are closely related to tumor onset, while increased copy number is associated with tumor progression [39, 40]. Therefore, the sensitivity to EGFR-TKIs between patients with coexisting EGFR mutation and amplification and patients with EGFR mutation may be different. However, our study proved that EGFR amplification is not associated with PFS. The discrepancy might be driven by real clinical practice, which contains lots of confounding factors such as heterogeneity of individuals and different types of TKIs. And these confounding factors might offset the effect of EGFR amplification. The advantage of this study is that it is a prospective observational study from the real world. However, the findings of this study should to be explained interpreted cautiously. Because our findings are based on single-center study, a multi-center and larger sample size study should be warranted to further confirm our conclusion.

## Conclusion

Taken together, we found that EGFR amplification is not an independent prognostic factor for EGFR-mutated non-squamous NSCLC patients receiving first-line EGFR-TKI. However, EGFR amplification was a risk factor for progression in patients with brain metastasis. These findings indicated that EGFR amplification may be a prognosis factor in specific populations, and prospective identification of patients with EGFR amplification should be enrolled in clinical trials to further verify it.

## Data Availability

The datasets used and/or analyzed during the current study be deposited in Dryad Data. Link: https://datadryad.org/stash/share/ew_zc6wxeYIayI36cus1F_WkMweJpU43A-eKfW9G39w.

## Abbreviations

EGFR: Epidermal growth factor receptor
EGFR-TKIs: Epidermal growth factor receptor Tyrosine kinase inhibitors
TKIs: Tyrosine kinase inhibitors
PFS: Progression-free survival
NGS: Next-generation sequencing
OS: Overall survival
19del: EGFR exon 19 deletion
21-L858R: Exon 21 Leu858Arg
FFPE: Formalin-fixed paraffin-embedded
TNM: Tumor Node Metastasis
